# Changing paradigm in the treatment of amyloidosis: From disease-modifying drugs to anti-fibril therapy

**DOI:** 10.3389/fcvm.2022.1073503

**Published:** 2022-12-20

**Authors:** C. Cristina Quarta, Marianna Fontana, Thibaud Damy, Julia Catini, Damien Simoneau, Michele Mercuri, Pablo Garcia-Pavia, Mathew S. Maurer, Giovanni Palladini

**Affiliations:** ^1^Alexion, AstraZeneca Rare Disease, Boston, MA, United States; ^2^Royal Free London NHS Foundation Trust, London, United Kingdom; ^3^University Hospital Henri Mondor, Creteil, France; ^4^Hospital Universitario Puerta de Hierro Majadahonda, Instituto de Investigación Sanitaria Puerta de Hierro Segovia de Arana (IDIPHISA), Centro de Investigación Biomédica en Red Enfermedades Cardiovasulares (CIBERCV), Madrid, Spain; ^5^Centro Nacional de Investigaciones Cardiovasculares (CNIC), Madrid, Spain; ^6^Columbia University Irving Medical Center, New York Presbyterian Hospital, New York, NY, United States; ^7^Department of Molecular Medicine, University of Pavia, and Amyloidosis Research and Treatment Center, Foundation “Istituto di Ricovero e Cura a Carattere Scientifico (IRCCS) Policlinico San Matteo”, Pavia, Italy

**Keywords:** cardiac light chain amyloidosis, cardiac amyloidosis (CA), cardiac amyloidosis—ATTR, standard of care (SoC), treatment gaps, future treatments

## Abstract

Cardiac amyloidosis is a rare, debilitating, and usually fatal disease increasingly recognized in clinical practice despite patients presenting with non-specific symptoms of cardiomyopathy. The current standard of care (SoC) focuses on preventing further amyloid formation and deposition, either with anti-plasma cell dyscrasia (anti-PCD) therapies in light-chain (AL) amyloidosis or stabilizers of transthyretin (TTR) in transthyretin amyloidosis (ATTR). The SoC is supplemented by therapies to treat the complications arising from organ dysfunction; for example, heart failure, arrhythmia, and proteinuria. Advancements in treatments have improved patient survival, especially for those whose disease is detected and for whom treatment is initiated at an early stage. However, there still are many unmet medical needs, particularly for patients with severe disease for whom morbidity and mortality remain high. There currently are no approved treatments to reverse amyloid infiltration and deplete the amyloid fibrils already deposited in organs, which can continue to cause progressive dysfunction. Anti-fibril therapies aimed at removing the deposited fibrils are being investigated for safety and efficacy in improving outcomes for patients with severe disease. However, there is no clinical evidence yet that removing deposited amyloid fibrils will improve organ function, thereby improving quality of life or extending life. Nevertheless, anti-fibril therapies are actively being investigated in clinical trials to evaluate their ability to complement and synergize with current SoC.

## Introduction

Amyloidosis is a rare and debilitating disease caused by misfolded proteins that self-aggregate into amyloid fibrils and deposit into various organs (1, 2). Cardiac amyloidosis (CA) results when amyloid fibrils deposit in the interstitial spaces of the myocardium (1, 2). CA is associated with long delays in diagnosis and may be followed by short period between diagnosis and death, especially among patients with advanced disease (3). Progressive deposition of amyloid fibrils in the myocardium results in a loss of cardiac architecture and function, leading to poorer quality of life, increased hospitalizations, and death (1, 2, 4, 5). In light chain (AL) amyloidosis, the circulating amyloid precursor also contributes to cardiac dysfunction through direct toxicity (6). Although the epidemiology of CA is not fully established, it is believed that CA is underrepresented as a cause of heart failure (7, 8).

**Figure 1:**
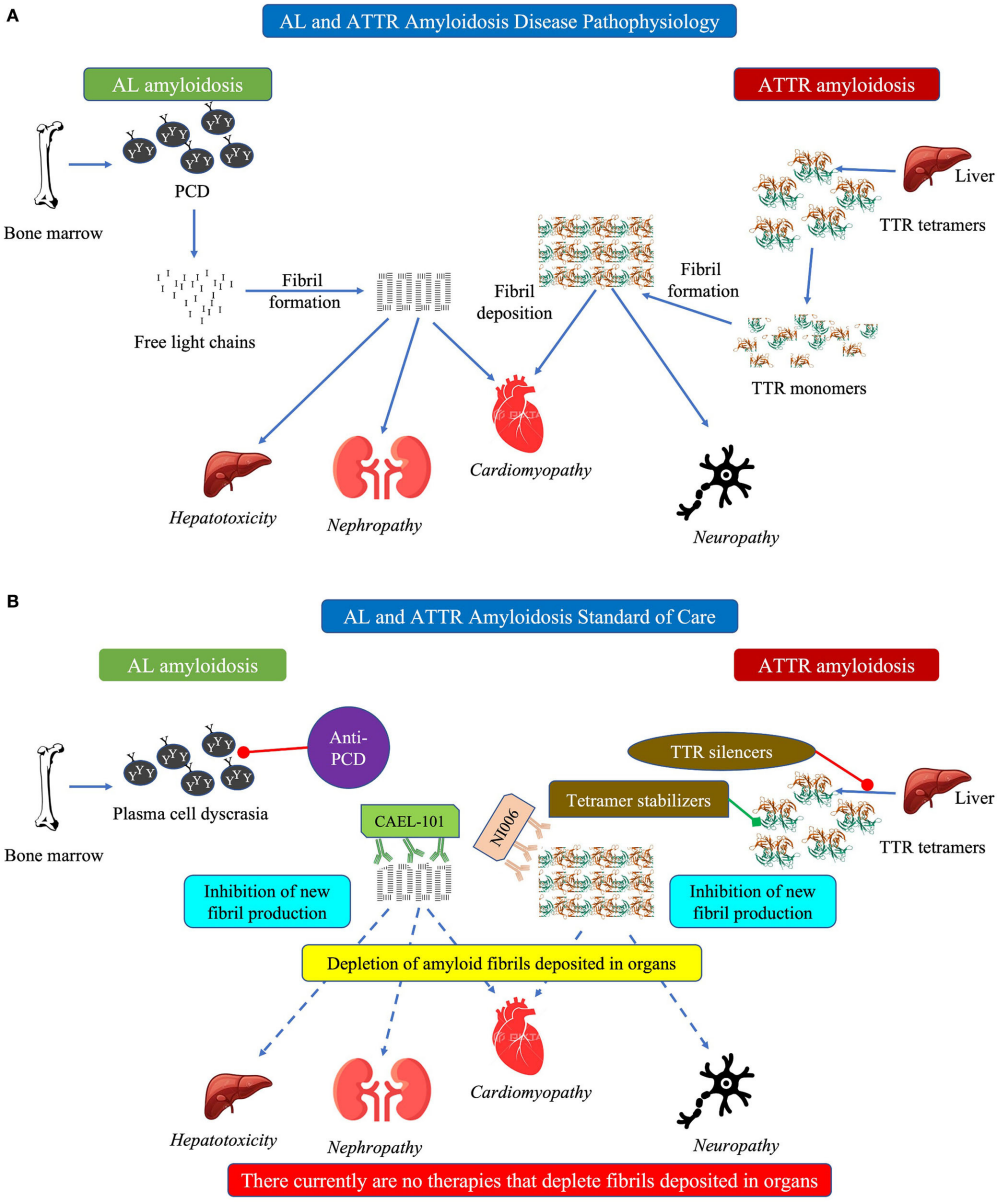
**(A)** Schematic diagram showing pathophysiology of cardiac amyloidosis and **(B)** schematic diagram showing current standard of care in cardiac amyloidosis. In **(A)** this schematic figure shows the pathophysiology of how both AL (left side) and ATTR (right side) amyloidosis can cause cardiomyopathy. In **(B)** this schematic figure shows the current standard of care for AL amyloidosis (anti-PCD) and ATTR amyloidosis (TTR silencers, tetramer stabilizers). Text in italics indicate manifestations of the disease. AL, light chain amyloidosis; ATTR, transthyretin amyloidosis; PCD, plasma cell dyscrasia; TTR, transthyretin.

The 2 main forms of CA are AL and transthyretin (TTR) amyloidosis (ATTR; Figure 1A) (1–4, 6–8). The fibrils in AL amyloidosis consist of misfolded immunoglobulin light chains resulting from clonal B-cell proliferation or plasma cell dyscrasia (PCD) originating in the bone marrow (1–4, 6–8). The fibrils in ATTR amyloidosis consist of misfolded TTR forming due to dissociation of either the wild-type protein (ATTRwt) or facilitated by mutations in the TTR gene (ATTRv) (1–4, 6–8). Both forms of CA may be difficult to diagnose due to non-specific symptoms and overlap with other cardiomyopathies causing delayed initiation of treatment and, consequently, poorer prognosis (9, 10). Survival was significantly better among patients with AL amyloidosis diagnosed in < 6 months from symptom onset with >52% of patients surviving over the 5-year period of the study than for those whose diagnosis took longer who also had significantly increased risk of death as shown by >63% of patients dying during the study period (9). Undiagnosed and delayed diagnosis of CA results in high morbidity and high mortality whereas early diagnosis has both clinical and quality of life (QoL) benefits for patients with AL, ATTRwt, or ATTRv CA (10–12). Using a disease simulation model, early diagnosis and timely treatment have been shown to extend the calculated life expectancy from the onset of symptoms by more than 5 and more than 7 years among patients with ATTRwt and ATTRv amyloidosis, respectively (12).

## Standard of care and limitations

Treatments for AL, ATTRwt, and ATTRv amyloidosis are very different. Thus, it is critical to identify the amyloidosis type and characterize the fibrils prior to treatment initiation to ensure patients receive the correct treatment (7, 8, 13). Treatment of CA is largely risk-adapted based on the disease burden and stratification of patients. For both AL and ATTR amyloidosis, the standard of care (SoC) focuses on preventing further generation and deposition of amyloid fibrils combined with supportive care (Figure 1B) (1, 7, 8, 13, 14). In addition, both AL and ATTR amyloidosis need a multidisciplinary team of specialists, specific to each patient, to manage the primary disease and the myriad comorbidities that occur (15–17).

### AL amyloidosis

The basis of SoC is increased overall survival and improved organ function when amyloidogenic light chain synthesis is suppressed or stopped (18). Therefore, the focus of current, risk-adapted therapy is to prevent more amyloid fibril generation (1, 7, 8, 13, 14). The source of amyloidogenic light chains is a clonal expansion of a plasma cell, similar to multiple myeloma (1, 3, 8, 13, 14). Therefore, most therapeutic agents currently used to treat AL amyloidosis are those with proven efficacy in treating multiple myeloma (19). Validated criteria for hematologic response, classified as complete response (CR), very good partial response (VGPR), and partial response (PR), and organ response have been published previously (20). The main options are autologous stem cell transplant (ASCT) or anti-PCD chemotherapy/immunotherapy to eliminate the underlying PCD (21, 22). However, very few patients are candidates for ASCT as strict eligibility criteria, including age < 70 years, low troponin and natriuretic peptides values, preserved cardiac, hepatic and renal function, are essential to reduce transplant-related mortality (22–27). Many patients eligible for ASCT benefit from induction therapy with anti-PCD regimens to prepare them for transplant and to improve outcomes (23, 25, 28). Patients obtaining good hematologic response with induction therapy may not need ASCT (26, 29).

For patients who are ineligible for ASCT, and those who decline the procedure, anti-PCD chemotherapy is the only option (23, 28). Current guidelines recommend a combination of cyclophosphamide, bortezomib, dexamethasone (CyBorD), and daratumumab as first-line therapy for newly diagnosed patients with AL amyloidosis (27, 28). Cyclophosphamide is an alkylating agent that causes damage to DNA strands resulting in apoptosis of the cell (30). Bortezomib is a proteasome inhibitor (31). Proteasomes are a multi-subunit enzyme complexes found in large numbers in the cell and are involved in reducing proteotoxicity and regulating proteins that control cell-cycle progression and apoptosis (32, 33). Inhibition of the catalytic core of proteasomes results in accumulation of ubiquitinated proteins and cellular apoptosis (31). Amyloid-generating plasma cells are particularly sensitive to proteasome inhibition because they rely on the proteasome to reduce the toxic effects of the amyloidogenic light chains and prevent apoptosis (34). Dexamethasone induces cellular apoptosis *via* the nuclear glucocorticoid receptor (35). However, dexamethasone use is associated with an increased risk of a cardiac event and death among patients with severe CA (stage IIIb per the European modification of the Mayo 2004 staging system) (36, 37). There is the potential need for monitoring in an intensive care unit for all high-risk patients, e.g., those in Stage IIIb and IV, receiving chemotherapy (38, 39). Daratumumab is a monoclonal antibody (mAb) that binds to CD38, a transmembrane glycoprotein expressed on the surface of plasma cells, causing apoptosis (40). It is the only agent specifically approved for treating AL amyloidosis when administered with CyBorD. Efficacy of CyBorD-daratumumab is very high, with 78% of patients achieving significant hematologic response, defined CR or VGPR (41–43). In a small group of patients with AL amyloidosis, median survival was 655 days for those treated with CyBorD (*n* = 15) compared with 178 days for those treated with melphalan-dexamethasone (*n* = 10) (44).

However, a survey of patients with AL amyloidosis reported more than 30% reducing treatments and more than 20% discontinuing at least 1 treatment due to adverse events (AE), requiring patients to receive other drug combinations (25, 45). In regions where daratumumab or bortezomib are not available, other combinations of alkylating agents, steroids, and immunomodulatory agents often are used as first-line therapy (46). Patients who are refractory to first-line anti-PCD therapy or who relapse are treated with immunomodulatory agents (e.g., thalidomide, lenalidomide, and pomalidomide), usually in combination with dexamethasone, to overcome resistance to alkylating agents and proteasome inhibitors (23, 47). Regardless of the anti-PCD treatment regimen, all patients require comprehensive supportive care from diagnosis onward to maintain organ function as best possible (48).

Of note, all anti-PCD therapeutic agents are chemotherapeutic agents that cause cell death and consequently can have relevant toxicity. Alkylating agents like cyclophosphamide and melphalan can have severe side effects, including hematopoietic, gastrointestinal, hepatic, gonadal, pulmonary, renal, cardiac, and neural toxicity (30). Treatment with bortezomib can result in peripheral neuropathy (49). The combination of bortezomib and dexamethasone can increase plasma levels of N-terminal pro-brain natriuretic peptide (NT-proBNP), a biomarker for cardiomyopathy, and risk of death especially among patients with advanced disease (36, 37). Immunomodulators are associated with increased cardiomyopathy, thromboembolic complications, myelosuppression, immunosuppression, renal failure, and may aggravate heart failure (18, 23, 25, 47, 50). Despite recent advances, only about 50% of patients achieve complete hematologic response with the currently available therapies. Unless effective rescue treatment is employed, the disease can continue to progress in many patients, particularly those who do not attain at least hematologic VGPR, indicating a huge therapeutic gap. Isatuximab, an anti-CD38 antibody similar to daratumumab, is under investigation to treat the PCD underlying AL amyloidosis (51). However, like all other anti-PCD therapies, it does not address removal of the fibrils already deposited in organs (51).

### ATTR amyloidosis

The current SoC for ATTR amyloidosis involves disease-modifying therapy to address the underlying disease, symptomatic therapy to manage cardiovascular and neurologic complications, supportive care, and genetic counseling (52–54). The goal of specific treatment in ATTR amyloidosis is to stabilize the TTR tetramer or stop amyloid fibril production (55).

The liver produces about 95% of TTR measured in the serum (52, 55). Hence, liver transplantation historically has been the first-line therapy to eliminate the main source of amyloidogenic TTR (52, 53, 55, 56). However, progression of CA after liver transplantation limits its utility, especially among patients with advanced disease (53, 55–57). This may be due to the continued presence of small amyloid fibril fragments that stimulate the aggregation of larger, pathogenic fibrils—a process termed “amyloid seeding” (58).

### Silencers

TTR expression can also be decreased pharmacologically using agents that “silence” or block the synthesis of the TTR protein (Figure 1B) (59). Antisense oligonucleotides (ASO), such as inotersen, are single-stranded deoxyribonucleotide strands that are complementary to the mRNA target and block protein production of the target, TTR in this case (59, 60). Inotersen, administered subcutaneously, stabilized cardiac symptoms in patients with ATTR cardiomyopathy (ATTR-CM) (61). Whereas treatment with inotersen significantly improves neurological symptoms, in rare cases it can cause severe thrombocytopenia and glomerulonephritis resulting in a boxed warning (53, 56, 61, 62). To prescribe inotersen in the USA, physicians must be trained and certified in Risk Evaluation and Mitigation Strategy (REMS) of the drug and their patients must be enrolled in the REMS program and undergo regular monitoring.

Small interfering RNA (siRNA), such as patisiran, are a class of short double-stranded non-coding RNA molecules that recognize and degrade target mRNA, TTR mRNA in this case (59, 63, 64). Treatment with patisiran, currently only approved for treating ATTR polyneuropathy (ATTR-PN), also might be associated with cardiac amyloid regression in a proportion of patients, as evidenced by reduced extracellular volume and disease stabilization with significant differences in NT-proBNP, left ventricular wall thickness, global longitudinal strain, and cardiac output. In these patients, changes also were associated with improved overall survival and lower cardiovascular-related hospitalizations compared with placebo (65, 66). In the APOLLO-B study (NCT03997383), compared with placebo patisiran significantly improved the functional capacity, measured with 6-min walk test (6MWT), and quality of life (QoL) of patients with ATTR-CM at 12 months with no additional safety signals (reported at XVIII International Symposium on Amyloidosis, 4–8 September 2022, Heidelberg, Germany). However, there were no statistically significant benefits observed in composite secondary endpoints, including all-cause mortality. Although patisiran has fewer concerns about AE than inotersen, it needs to be administered by a trained healthcare professional and patients are exposed for long periods to corticosteroids and antihistamines to limit infusion reactions (67).

Since a normal physiological function of TTR is transporting vitamin A, reduction of TTR, due to either inotersen or patisiran, results in severe vitamin A depletion and requires daily supplementation to maintain normal levels (53, 56, 61, 62). Both drugs show improvement among patients with low to moderate disease burden. Recent data suggest patisiran also may have benefits among patients with ATTR-CM.

Vutrisiran is a second-generation siRNA formulation of patisiran and can block the expression of both ATTRwt and ATTRv genes (68). It has been approved for treatment of polyneuropathy. Compared with placebo, vutrisiran was shown to reduce serum levels of NT-proBNP, improve some echocardiographic parameters, and improve scintigraphy tracer uptake in ATTRv patients with polyneuropathy. It is currently under investigation for treatment of patients with ATTR-CM (NCT04153149).

Eplontersen is a ligand conjugated ASO with the same primary sequence as inotersen (69). Conjugation with ligand facilitates targeted uptake of the drug by hepatocytes, which has the potential for greater efficacy and lower toxicity than the unconjugated drug, inotersen (69). Eplontersen is reported to significantly lower TTR levels from baseline and be well tolerated. It is under investigation for treatment of both ATTR-CM and ATTR-PN. In the NEURO-TTRansform trial (NCT04136184), compared with placebo eplontersen slowed the progression of neuropathic disease and improved QoL among patients with hereditary ATTR-PN (reported at XVIII International Symposium on Amyloidosis). No specific safety concerns were reported.

### Stabilizers

Another approach to treating ATTR amyloidosis is to stabilize the TTR tetramer protein complex, thereby preventing its dissociation into amyloidogenic TTR monomers and oligomers (Figure 1B) (70). Native TTR tetramer stabilization requires both thyroxine-binding pockets of the TTR tetramer to be occupied, thus requiring high concentrations of TTR stabilizers to prevent its dissociation (66).

Diflunisal, an oral non-steroidal anti-inflammatory drug (NSAID), stabilizes TTR tetramers (52). However, long-term use may be associated with increased fluid retention leading to heart failure, gastritis, peptic ulcer disease, and worsening of renal dysfunction (71). Therefore, diflunisal should be used with caution in older adults and in patients with severe congestive heart disease. Moreover, renal insufficiency limits its use in patients with ATTR-CM or ATTR-PN with cardiac or renal impairment requiring careful patient selection and management of drug-associated AEs (52).

Tafamidis is an orally bioavailable agent, and the only one approved to treat ATTR-CM (72). It occupies the thyroxine-binding sites in wild-type and several variants of TTR with high affinity and selectivity, thereby preventing their dissociation (73). Compared with placebo, treatment with tafamidis was associated with TTR stabilization in almost all patients, significantly lower all-cause mortality, lower rate of cardiovascular-related hospitalizations, and less decrease in 6MWT functional capacity indicating that tafamidis stabilized disease, delayed disease progression, and slowed decline in patient QoL (72, 74). However, a pre-specified subgroup of patients with New York Heart Association (NYHA) class III heart failure, representative of patients with advanced disease, had a higher rate of cardiovascular-related hospitalizations compared with placebo, i.e., an inverse relationship between effectiveness and NYHA class (72). However, improved survival was observed at 5-year follow-up among patients in NYHA class III compared with those who received placebo initially (reported at European Society of Cardiology Heart Failure Congress, 26–29 May 2022, Barcelona, Spain). Patients in NYHA class IV, representative of very advanced disease, were excluded from this trial. In all clinical studies, tafamidis demonstrated an appropriate benefit-to-risk ratio.

Acoramidis (AG10/ALXN2060) is a new TTR stabilizer under investigation for treatment of ATTR amyloidosis (75). Acoramidis binds TTR with greater selectivity than either tafamidis or diflunisal and increases serum levels of TTR tetramers and is well tolerated (75–77). In the ongoing open-label extension of the phase 2 trial (NCT03536767), serum TTR levels were below the lower limit of normal in 40.4% of patients and there was a median decrease from baseline of 479 pg/mL in serum NT-proBNP levels (78). Two randomized, double-blind, placebo-controlled, phase 3 studies (NCT03860935 and NCT04622046) are in progress to determine the efficacy and safety of acoramidis in patients with ATTR-CM.

Another stabilizer under investigation for treatment of ATTR amyloidosis is tolcapone (79). Like acoramidis, tolcapone binds TTR with greater selectivity than either tafamidis or diflunisal and increases serum levels of TTR tetramers (79, 80). However, tolcapone has a boxed warning for potentially fatal acute fulminant liver failure and is not suggested for patients with ATTR-CM (77).

## Future anti-fibril therapies

**Figure 2:**
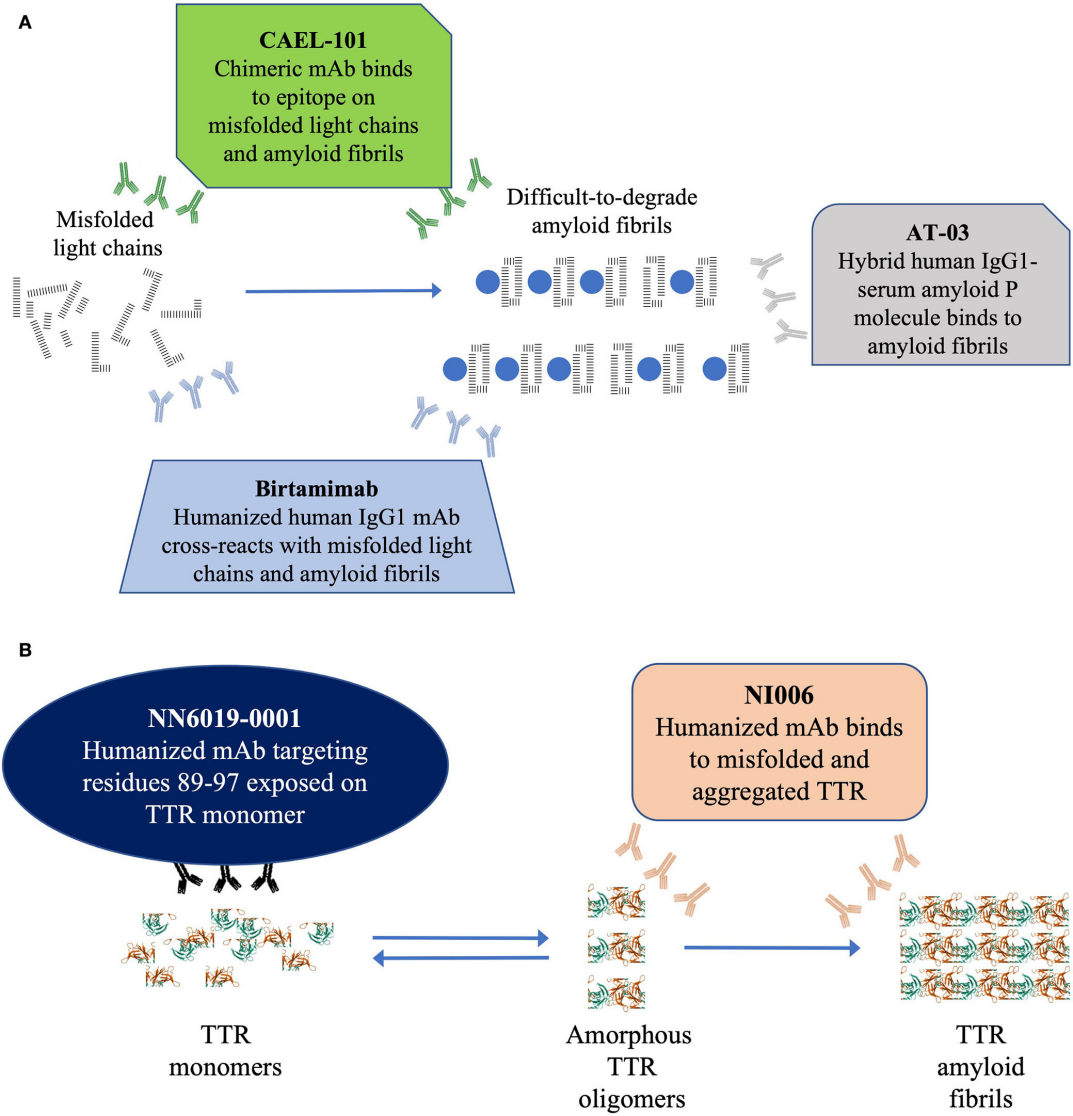
**(A)** Schematic diagram depicting the 3 anti-fibril mAb therapies currently under investigation for AL amyloidosis and **(B)** Schematic diagram depicting the 2 anti-fibril mAb therapies currently under investigation for ATTR amyloidosis. In **(A)** this schematic figure shows the 3 mAbs currently under investigation for depletion of light-chain fibrils in AL amyloidosis. In **(B)** this schematic figure shows the 2 mAbs currently under investigation for depletion of TTR fibrils in ATTR amyloidosis. AL, light chain amyloidosis; IgG1, immunoglobulin G1; mAb, monoclonal antibody.

Despite advances in treatment options, there are as yet no approved treatments for removal of amyloid fibrils already deposited in the organs, especially the heart (61, 81). These fibrils can continue to cause progressive damage to the organs resulting in death. The current hypothesis is that removal or depletion of these amyloid fibrils will decrease organ damage and restore function, particularly that of the heart resulting in improved survival (Figures 2A,B).

### AL amyloidosis

There currently are 3 mAbs, birtamimab, CAEL-101, and AT-03 (Figure 2A), under investigation as anti-fibril agents (82–84). It is hoped that these antibodies provide direct proof of concept by depleting the deposits of light chain amyloid fibrils from organs improving their function.

Birtamimab, a fully humanized mAb developed to recognize a cryptic epitope on serum amyloid A protein, cross reacts with light chain amyloid fibrils and activates macrophage-mediated degradation and clearance of the fibrils (83). In a phase 1/2 trial, birtamimab was well tolerated at all doses administered up to 24 mg/kg (85). In the phase 2b PRONTO trial (NCT02632786), birtamimab failed to improve cardiac response, 6MWT, and NT-proBNP levels in previously treated patients with AL amyloidosis (86). Furthermore, a futility analysis of the phase 3 VITAL (NCT02312206) trial showed that birtamimab did not reduce all-cause mortality in newly diagnosed patients resulting in termination of the trial (87). However, a *post-hoc* analyses showed promising results among patients in Mayo 2012 Stage IV (85, 87, 88). A double-blind, placebo-controlled, phase 3 trial (AFFIRM-AL; NCT04973137) is currently recruiting patients to confirm these results (89).

CAEL-101, a chimeric mAb developed to recognize a cryptic epitope on immunoglobulin light chains, binds to misfolded free light chains and amyloid fibrils deposited in organs (90–92). In phase 1 trials, CAEL-101 demonstrated reductions in biomarkers of cardiomyopathy and nephropathy (93–95). The ongoing phase 2 trial demonstrated that CAEL-101 was well tolerated when administered with anti-PCD therapy that included daratumumab or as monotherapy after cessation of anti-PCD therapy. There are 2 concurrent randomized, double-blind, placebo-controlled, phase 3 trials actively recruiting patients with advanced cardiac disease 2015 European Modification of Mayo 2004 Stages IIIA (NCT04512235) and IIIB (NCT04504825) (96).

AT-03, a hybrid human mAb against serum amyloid P protein, binds all types of amyloid fibrils (84). AT-03 has recently completed a phase 1 biodistribution study (NCT05201911), the results of which have not yet been reported.

### ATTR amyloidosis

There currently are 2 mAbs under investigation to deplete or remove TTR amyloid fibrils, NI006 and NN6019-0001 (formerly known as PRX004; Figure 2B) (73, 97). It is hoped that these antibodies will provide direct proof of concept by depleting TTR amyloid fibril deposits from organs and improving their function.

NI006 is a humanized mAb that selectively binds to a cryptic epitope that is exposed in misfolded TTR oligomers and aggregated TTR fibrils (97). In preclinical studies, NI006 bound with high affinity to both ATTRwt and ATTRv amyloid fibrils and facilitated their elimination *via* activation of phagocytic cells. A phase 1 study (NCT04360434) is in progress to determine the dosage and safety of NI006 in patients with ATTR-CM.

NN6019-0001 binds to a cryptic epitope that is exposed when TTR tetramers dissociate into monomers (73). NN6019-0001 binds and neutralizes the various prefibrillar species of TTR and prevents the formation of new amyloid fibrils (73). In phase 1 trials, NN6019-0001 was well tolerated at all doses and showed improvement in both global longitudinal strain and neurologic symptoms (reported at XVIII International Symposium on Amyloidosis, 4–8 September 2022, Heidelberg, Germany). A placebo-controlled, phase 2 study (NCT05442047) is currently recruiting patients to determine the efficacy and safety of NN6019-0001 at 10 and 60 mg/kg.

## Conclusions

Although there have been considerable advances in the treatment of CA, there are still opportunities for improvement. Overall CR among patients with AL amyloidosis still hovers around 50% and does not always translate into organ response. Despite advances, disease continues to progress for many patients. Current therapies to treat both AL and ATTR amyloidosis are focused on eliminating or stabilizing the source of the amyloidogenic protein. However, there are no approved therapies that deplete or eliminate already deposited amyloid fibrils, which can continue to cause progressive organ damage, especially the heart, causing early death. There are promising studies focusing on amyloid fibril depletion in both forms of CA that are expected to add to the armamentarium available and to elucidate whether removal of preexisting fibrils will improve survival and QoL of patients.

## Author contributions

CCQ was invited to contribute. All authors provided critical input into the concept and content of this review article. All authors approved the submission of the final article.
